# Screening Method Based on Walking Plantar Impulse for Detecting Musculoskeletal Senescence and Injury

**DOI:** 10.1371/journal.pone.0083839

**Published:** 2013-12-30

**Authors:** Yifang Fan, Yubo Fan, Zhiyu Li, Tony Newman, Changsheng Lv, Yi Zhou

**Affiliations:** 1 Center for Scientific Research, Guangzhou Institute of Physical Education, Guangzhou, P.R. China; 2 Key Laboratory for Biomechanics and Mechanobiology of Ministry of Education, School of Biological Science and Medical Engineering, Beihang University, Beijing, P.R. China; 3 College of Foreign Studies, Jinan University, Guangzhou, P.R. China; Scientific Institute Foundation Santa Lucia, Italy

## Abstract

No consensus has been reached on how musculoskeletal system injuries or aging can be explained by a walking plantar impulse. We standardize the plantar impulse by defining a principal axis of plantar impulse. Based upon this standardized plantar impulse, two indexes are presented: plantar pressure record time series and plantar-impulse distribution along the principal axis of plantar impulse. These indexes are applied to analyze the plantar impulse collected by plantar pressure plates from three sources: Achilles tendon ruptures; elderly people (ages 62–71); and young people (ages 19–23). Our findings reveal that plantar impulse distribution curves for Achilles tendon ruptures change irregularly with subjects’ walking speed changes. When comparing distribution curves of the young, we see a significant difference in the elderly subjects’ phalanges plantar pressure record time series. This verifies our hypothesis that a plantar impulse can function as a means to assess and evaluate musculoskeletal system injuries and aging.

## Introduction

A walking plantar impulse expresses the action of the foot on a support surface [1], [2]. Titianova et al. developed the pressure sensor system to acquire plantar impulse information [3]. But walking plantar impulse analysis is not widely used in clinical diagnosis or rehabilitation assessment [4]–[7]. The lack of convincing and effective representation of conditions such as musculoskeletal system injuries or aging has bottlenecked the wide application of a plantar impulse. The arrival of an aging society [8], [9] and the increase of sports injuries [10], [11] call for more approaches to better assess and evaluate these two groups.

When walking, the interaction between a foot and its support surface - obtained from the plantar pressure measurement device - can be differentiated by pressure sensors into the force of unit area. A pressure sensor will not only capture the interaction force at a specific instant, but it will also capture the initial and terminal time of the interaction process. The combination of plantar initial contact ground (ICG) and terminal contact ground (TCG) time is called the pressure record time series (PRTS) and the resulting pressure value reading from pressure sensors is called the instantaneous pressure [12]. When walking bare-footedly, at stance phase, the temporal integration of the instantaneous pressure is called unit impulse, and the sum of each unit impulse that the foot makes contact with is a plantar impulse.

The walking-footprint progression angle, walking-footprint size, plantar pressure and plantar impulse are influenced not only by individual body shape and structure [13], but also by walking speed [14], [15], and walking plantar impulse. The definition of an average value [16], [17] indicates that average plantar impulse can generalize the whole plantar impulse and the average PRTS can reflect the characteristics of how the plantar impulse is shaped. Healthy people’s many indexes (mean) are used to identify abnormalities in clinical and rehabilitation medicine, which means that when differences between plantar impulses are eliminated, to such an extent that they can meet the requirements of average value, it solves the critical problem of plantar impulse analysis.

In this study, a distribution of plantar impulse is defined as a physical quantity, which is the sum of all the products formed by multiplying the magnitude of each unit impulse by the square of its distance to the plantar impulse center. Mathematical analysis indicates that this quantity has a principal axis, which is called the plantar-impulse principal axis (PIPA). Just like the uniqueness of a principal axis of inertia of an asymmetrically shaped and anisotropically structured object [18], the PIPA is also unique. This means that we can use the PIPA to standardize the plantar impulse, to calculate the initial and terminal contact ground time series along the PIPA, to rate plantar-impulse distribution length by percentage, to normalize stance time and to establish a PRTS index along the PIPA. After rating the length and width of the processed plantar impulse by percentage, every unit impulse is divided by total impulse. Thus, we establish a plantar-impulse distribution index along the PIPA.

We assume that a plantar impulse contains information about musculoskeletal system injuries and aging: function affects behavior. If our hypothesis holds, then it is possible to build an evaluation index to reflect musculoskeletal system injuries and aging based on walking plantar impulse. Our comparative analysis of the elderly subjects’ free gait with the young subjects’ free, fast and slow gait shows that the PRTS index along PIPA can reflect the musculoskeletal system’s senescence, while that of different speeds from the Achilles tendon ruptures (ATRs) and from the young shows that plantar-impulse distribution index along the PIPA can reflect the musculoskeletal system’s injury, confirming our hypothesis.

## Materials and Methods

### Ethics Statement

The study received approval from the Ethical Committee of Guangzhou Institute of Physical Education. The subjects provided fully informed consent to participate in this study by signing a written consent form.

### Walking Plantar Pressure Test Equipment

Walking plantar pressure measuring equipment: Zebris FDM System - Gait Analysis (Long platform). Platform: 56(W)×608(L) cm, with an additional 1.2 m at each end of the platform; sensor intensity: 1 sensor/cm^2^; sampling rate: 100 Hz. software: WinFDM. Test results were output as text file (unit plantar pressure data at every instant of time) to be used in this study. Image processing was done by MATLAB and background (i.e. foot’s three-dimensional image) was done by Mimics.

### Test Subjects and Requirements

Our subjects were divided into three groups. The first group: twenty healthy subjects - 10 male subjects: 21.1±1.31 year, 1.72±0.64 m, 61.8±8.3 kg and 10 female subjects: 21.7±1.3 year, 1.61±0.57 m, 51.2±7.6 kg. The second group: thirty healthy elderly subjects - 14 male subjects: 68.1±3.31 year, 1.69±0.85 m, 63.8±8.3 kg and 16 female subjects: 66.5±3.1 year, 1.59±0.67 m, 50.2±6.6 kg. For their basic gait information, see Table S1. The third group: seven male subjects with ATRs: see Table 1 for details. For these seven subjects’ basic gait information, see Table S2.

**Table 1:** Detailed information of ATRs.

Subject	Height	Weight	Age	Cause of ATRs
1	171	78	60	with right foot ATR when playing basketball in January, 2000
2	175	83	41	with left foot ATR when playing basketball in July, 2002
3	165	69	45	with right foot ATR when playing basketball in July, 2003
4	160	65	44	with left foot ATR in gymnastics training in May, 2005
5	166	65	46	with left foot ATR when playing basketball in April, 2007
6	180	78	34	with left foot ATR when playing football in April, 2011
7	167	75	48	with right foot ATR when playing basketball in June, 2011

Units in the table: height: cm; weight: kg; age: year.

A questionnaire was given to the candidates to exclude those with lower extremity ligament injury history. Each subject’s annual medical check-up report was screened to exclude those with disease or trauma in their nervous and/or musculoskeletal system. Medical reports were provided by the hospital to seven subjects with ATRs.

When measuring at different speeds, no metronome or moving reference was used to intervene with the subjects’ walking speed. The subjects were asked to walk in their usual habit. In order to impress perceptual awareness upon the subjects, models of walking at free, fast and slow speeds were given before the test. (The values of the three speeds modeled by our demonstrator were 1.05 m/sec, 1.35 m/sec, 1.86 m/sec, respectively, and they were relatively stable.).

Before the test began, subjects were asked to stand barefooted after both feet had been sterilized with 75% ethyl alcohol. Then subjects began from standing with feet together (barefooted) at the start of the platform. When the subjects stood steadily (for about 3 seconds), the laboratory assistant gave instructions to begin walking. The equipment operator pressed the key of the equipment to collect the data. When the subjects stopped, they returned after being instructed to do so by the laboratory assistant. If the first step onto the platform was found to be incomplete, or if the subject walked off the platform, or if the gait seemed apparently nonsuccessive, the subject was asked to try again. Data that met our requirements were collected from their second step on the platform. Six successive steps from each subject were collected and then analyzed.

### Walking Plantar-impulse Principal Axis

A physical quantity is the sum of all the products formed by multiplying the magnitude of each unit impulse by the square of its distance to the impulse center. The plantar impulse is provided by Zebris FDM System Gait Analysis, with a component as:
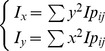
(1)where 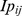 stands for the impulse of the interaction between foot and sensors, *i* and *j* stand for row and column number of sensor respectively. The unit impulse can be calculated by 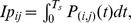 where 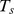 refers to stance time, 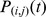 to instantaneous pressure value of sensor at instant *t*, and 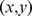 for position of 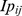 relative to plantar-impulse center, which is calculated by 
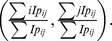


Set the angle displacement to be 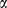, where plantar impulse rotates around the vertical axis that goes through plantar-impulse center. We can set up the following relation:
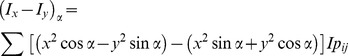
(2)


Differentiate Equation (2) and set
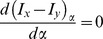



Then, we will obtain
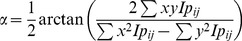
(3)


The shape of plantar-impulse distribution is asymmetrical, which leads to 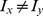 in Equation (1). Just like the uniqueness of principal axis of inertia of the asymmetrically shaped and anisotropically structured object, within the range of 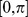, the limited rotation of the plantar impulse can always bring the result of 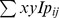 to be zero [18]. The axis that goes through plantar-impulse center is called the PIPA.

See File S1 for an example.

### Walking Plantar-impulse Distribution along PIPA

Along PIPA, the plantar impulse position 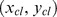 is calculated by the following equation:
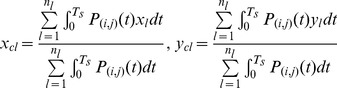
(4)where 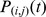 and 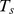 have the same definitions as those in Equation (1), and 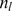 stands for the number of sensors (unit number of plantar-impulse distribution width) that interact with the foot at the foot length position *l*.

See File S1 for an example.

### PRTS along PIPA

According to gait characteristics, the progression of plantar contact (initial to terminal) with the ground is continuous, which can be represented by the fact that the same plantar position will initiate and terminate foot contact once, respectively, in one gait cycle [19]. That is to say, each plantar surface point contacts the ground only once during a step. If the plantar position (*x, y*) is set as 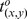 and 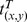, respectively, then the plantar ICG and TCG time series along PIPA will be
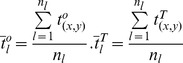
(5)where 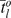 and 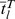 stand for the ICG and TCG time series at the position *l* along PIPA. 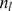 shares the same definition as that in Equation (4).

### Plantar Impulse along Foot Length

The plantar-impulse distribution along foot length position *l* will be:
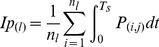
(6)where 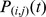, 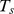 and 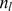 have the same definitions as those in Equation (4).

## Results and Discussion

**Figure 1:**
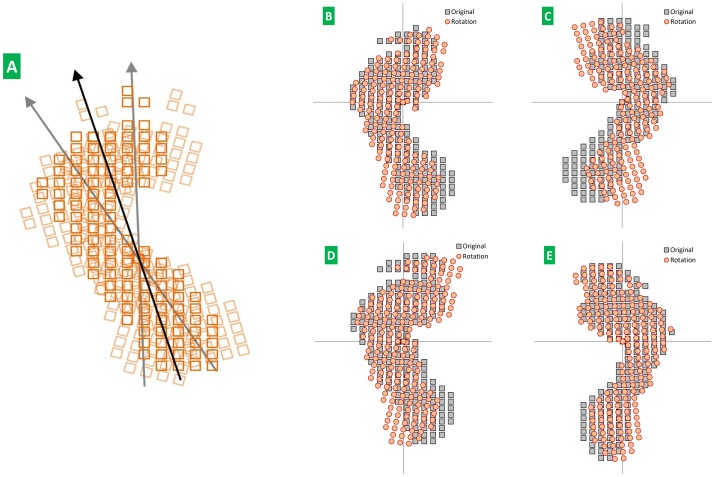
Plantar impulse fixed along PIPA. Fig. 1A Plantar-impulse rotation around the vertical axis that goes through plantar-impulse center. Fig. 1B First plantar impulse (left foot) before and after the plantar impulse is fixed. Fig. 1C Second plantar impulse (right foot) before and after the plantar impulse is fixed. Fig. 1D Third plantar impulse (left foot) before and after the plantar impulse is fixed. Fig. 1E Fourth plantar impulse (right foot) before and after the plantar impulse is fixed. In these figures, black stands for the original plantar impulse before it is fixed, red for the plantar-impulse position after it is fixed. A principal axis is identified after the first plantar impulse rotates 7.66 degree, the second −14.37 degrees, the third 10.25 degrees and the fourth −5.00 degrees.

Equation 1 is used to calculate the plantar-impulse center. Equation 3 is used to calculate each plantar-impulse rotation angle, which is applied by the plantar impulse to rotate around the vertical axis that goes through the plantar-impulse center. See Figure 1.

Figure 1 shows that when the plantar impulse is identified, its principal axis is unique, which guarantees the reliability of the standardization of the plantar impulse. In gait analysis, the walking plantar-impulse progression angle is a quantity related to walking direction and walking-footprint shape, which is a measurement value [13], [14]. This method, based upon the PIPA, provides an alternative analytical solution to standardize walking plantar impulse.

**Figure 2:**
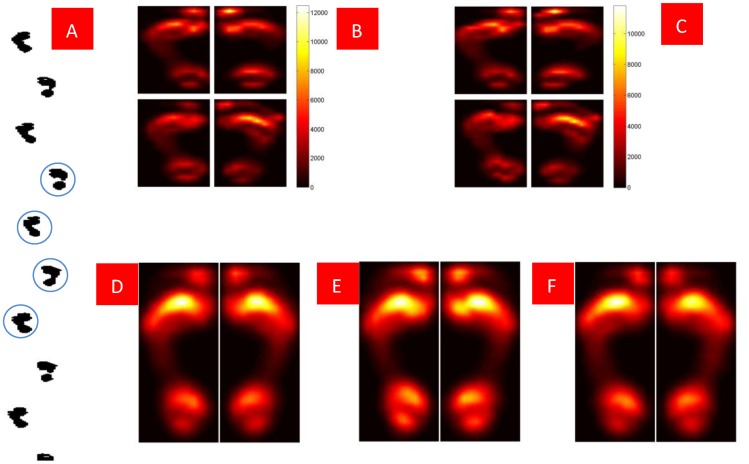
Standardization of plantar impulse. Fig. 2A Plantar impulse measured by Zebris FDM. Fig. 2B Four continuous plantar impulses. Fig. 2C Every plantar impulse is fixed by PIPA. Fig. 2D Plantar impulse of free gait after plantar impulse is standardized. Fig. 2E Plantar impulse at fast speed gait after plantar impulse is standardized. Fig. 2F Plantar impulse at slow speed gait after plantar impulse is standardized. Figs. 2B and 2C are from the same subject. Figs. 2D–F are the average plantar impulse from twenty healthy young subjects (each with six plantar impulses, three from left and right, respectively).

“Fixing” refers to the use of the physical quantity of PIPA to represent footprint progression angle [13]. When the plantar impulse is fixed, each walking plantar-impulse distribution length and width is rated by percentage, and the unit impulse is normalized by total impulse. The average plantar impulse at different speeds is shown in Figure 2.

Figure 2 shows the plantar impulse standardized by PIPA. Once the plantar-impulse distribution length and width are rated by percentage and the plantar unit impulse is normalized by total impulse, effects of individual difference in walking plantar-impulse progression angle, foot type and weight are eliminated [14]. Then the average plantar impulse from healthy young subjects at different walking speeds is obtained. Thus, the average plantar impulse can reflect the overall shape of a plantar impulse.

**Figure 3:**
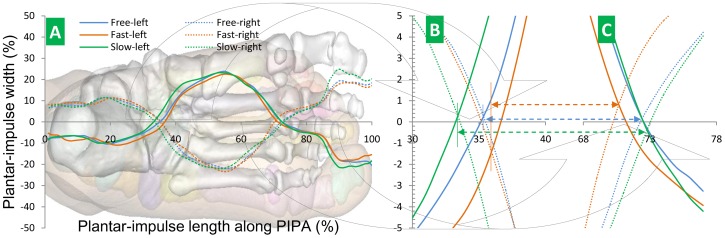
Distribution curve of plantar impulse. Fig. 3A Distribution curve of plantar impulse from the healthy young subjects at three walking speeds. Fig. 3B Intersection point (first intersection point) at heel. Fig. 3C Intersection point (second intersection point) at forefoot. The *x*-axis stands for the middle line in foot width.

Equation (4) is applied to calculate the plantar-impulse distribution curve position. The distribution curve of left and right foot is drawn. See Figure 3.

Figure 3 shows that based upon the average plantar impulse, the distribution curve of plantar impulse can be obtained along PIPA. The index can be used to analyze young subjects’ different walking speeds, which brings the result that the distribution curves of plantar-impulse form two intersection points – one at the heel and the other at the forefoot. When walking speed varies, the sequence of the first intersection point from the two distribution curves is slow-free-fast speed while that of the second intersection point is fast-free-slow. These sequences shorten the distance between two intersection points when walking speed increases. Our hypothesis that the plantar impulse reveals musculoskeletal system injuries is thus verified. The variation of intersection points changing with walking speed from the healthy subjects in this study can thus be used as an assessment method to identify musculoskeletal system injuries.

**Figure 4:**
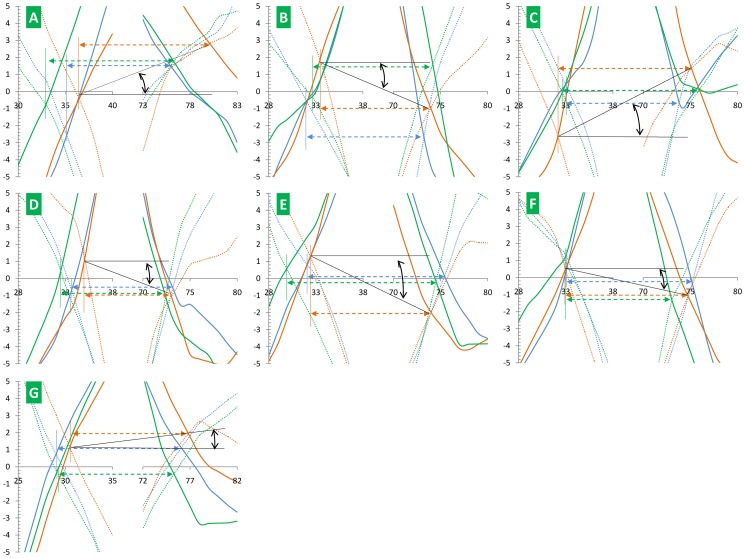
Distribution curve of plantar impulse from the ATRs. Figs. 4 A–G Intersection point (first intersection point) at heel and intersection point (second intersection point) at forefoot, where horizontal axis refers to the plantar-impulse length while the vertical one for plantar-impulse width.

The plantar impulse distribution curve (both left and right foot) from the ATRs is shown in Figure 4.

Figure 4 shows that at different speeds, the intersection point’s position of left/right plantar-impulse distribution curve from the ATRs, in comparison with those from the young subjects, has two characteristics in common: 1) the position sequence is irregular, which leads to the distance abnormality of two intersection points; 2) when walking at fast speed, the connection line between two intersection points, i.e. one that connects the first (at heel) and the second intersection point (at forefoot) and the sagittal axis of foot forms an angle: when the angle is greater than zero, it indicates a right foot ATR; when it is less than zero, a left foot ATR. But even the angle of the healthy subjects is not zero. This needs further exploration and discussion.

**Figure 5:**
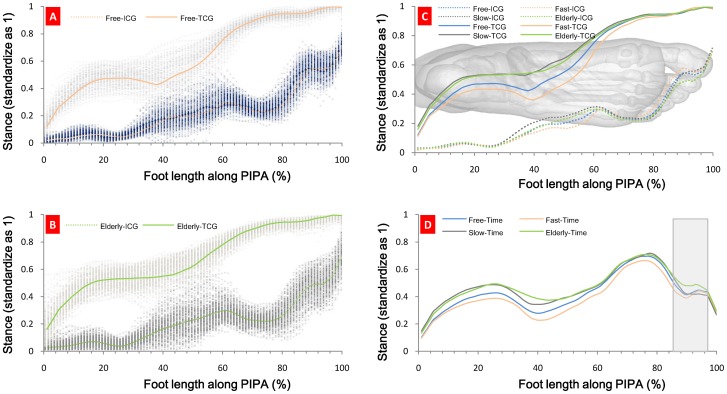
Plantar ICG and TCG time series along PIPA. Fig. 5A Plantar ICG and TCG time series along PIPA of healthy young subjects while walking at free gait. Fig. 5B Plantar ICG and TCG time series along PIPA of the healthy elderly subjects while walking at free gait. Fig. 5C Plantar ICG and TCG time series along PIPA of young subjects’ free, fast and slow speed gait and the healthy elderly subjects’ free gait. Fig. 5D Plantar stance time distribution along PIPA. Six continuous plantar impulses of the subject were initially collected (three from each foot). This average value can be employed as a toe ICG time series index along PIPA. We calculate the plantar stance time on PIPA using 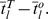

After the plantar impulse is fixed, Equation (5) is used to calculate the ICG and TCG time series along PIPA. Next, we rate the plantar-impulse distribution length by percentage and standardize the stance time as 1. The results are shown in Figure 5.

Figure 5 shows that plantar ICG and TCG time series are related to walking speed. For the healthy young, the effect from walking speed upon the distribution of ICG time series distribution is relatively small, especially in the heel, forefoot or toe; the effect is mainly upon the distribution of TCG time series, i.e. from heel to forefoot, the TCG time increases with an increase in walking speed; forefoot relative stance phase time increases with an increase in walking speed. This stance time distribution is consistent with the ground reaction force distribution at different walking speeds [20], indicating that this method to calculate PRTS along PIPA is reliable.

Figure 5 also shows the difference between the healthy elderly subjects’ ICG time series and that of the healthy young subjects. In order to demonstrate this difference, we analyze the ICG time series along PIPA from both the elderly and young. See Table 2.

**Table 2:** ICG time series along PIPA (Plantar-impulse length is rated by percentage).

%	Normal(n = 20)	Fast(n = 20)	Slow(n = 20)	Elderly(n = 30)
1	0.020±0.018	0.017±0.011	0.032±0.021	0.031±0.029
2	0.023±0.013	0.021±0.009	0.032±0.016	0.032±0.026
3	0.026±0.010	0.025±0.010	0.033±0.013	0.034±0.024
4	0.029±0.010	0.029±0.013	0.033±0.013	0.035±0.022
5	0.030±0.012	0.031±0.015	0.033±0.014	0.036±0.022
6	0.031±0.009	0.030±0.012	0.035±0.012	0.038±0.020
7	0.032±0.009	0.030±0.011	0.037±0.013	0.040±0.019
8	0.033±0.012	0.029±0.011	0.040±0.016	0.042±0.020
9	0.037±0.015	0.030±0.011	0.044±0.020	0.045±0.022
10	0.041±0.015	0.034±0.013	0.049±0.019	0.051±0.022
11	0.045±0.016	0.037±0.016	0.054±0.018	0.056±0.020
12	0.049±0.017	0.042±0.018	0.059±0.018	0.062±0.019
13	0.053±0.015	0.047±0.019	0.062±0.018	0.068±0.019
14	0.055±0.013	0.051±0.017	0.063±0.016	0.072±0.020
15	0.058±0.013	0.055±0.016	0.064±0.016	0.074±0.020
16	0.061±0.015	0.058±0.015	0.064±0.018	0.074±0.020
17	0.063±0.017	0.060±0.014	0.062±0.021	0.073±0.021
18	0.064±0.018	0.061±0.014	0.060±0.022	0.070±0.020
19	0.063±0.020	0.062±0.017	0.058±0.024	0.065±0.021
20	0.060±0.021	0.060±0.020	0.055±0.025	0.060±0.022
21	0.056±0.022	0.058±0.020	0.052±0.026	0.053±0.021
22	0.053±0.021	0.055±0.019	0.050±0.025	0.046±0.021
23	0.050±0.019	0.052±0.017	0.047±0.024	0.040±0.019
24	0.047±0.016	0.049±0.015	0.046±0.022	0.035±0.017
25	0.046±0.014	0.048±0.013	0.046±0.018	0.034±0.015
26	0.045±0.013	0.046±0.011	0.047±0.017	0.036±0.013♦
27	0.047±0.014	0.047±0.011	0.049±0.016	0.039±0.013
28	0.051±0.015	0.050±0.011	0.056±0.017	0.045±0.014
29	0.056±0.016	0.055±0.013	0.065±0.019	0.054±0.014
30	0.063±0.015	0.060±0.016	0.076±0.022▾	0.065±0.014
31	0.072±0.014	0.067±0.019	0.089±0.027▾	0.075±0.016
32	0.081±0.015	0.074±0.021	0.104±0.032▾	0.087±0.018
33	0.092±0.019	0.081±0.023	0.120±0.039▾	0.098±0.020
34	0.102±0.022	0.090±0.024	0.135±0.043▾	0.109±0.022
35	0.111±0.024	0.099±0.028	0.149±0.045▾	0.120±0.024
36	0.119±0.027	0.110±0.032	0.162±0.047▾	0.133±0.027
37	0.127±0.031	0.118±0.035	0.174±0.049▾	0.145±0.031
38	0.134±0.037	0.124±0.038	0.185±0.052▾	0.157±0.032
39	0.147±0.048	0.130±0.038	0.195±0.058▾	0.167±0.034
40	0.160±0.058	0.134±0.038	0.203±0.063▾	0.177±0.037
41	0.171±0.069	0.141±0.037	0.210±0.065	0.184±0.040
42	0.181±0.077	0.149±0.038	0.216±0.065	0.192±0.044
43	0.186±0.076	0.155±0.042	0.224±0.062	0.198±0.050
44	0.189±0.076	0.161±0.045	0.231±0.061	0.206±0.057
45	0.192±0.074	0.165±0.046	0.236±0.064	0.211±0.062
46	0.193±0.071	0.167±0.047	0.238±0.068	0.217±0.064
47	0.193±0.070	0.167±0.046	0.238±0.070	0.225±0.067
48	0.194±0.068	0.166±0.045	0.239±0.069	0.230±0.066
49	0.195±0.068	0.164±0.044	0.240±0.068	0.236±0.066
50	0.200±0.068	0.163±0.046	0.245±0.064	0.242±0.068
51	0.205±0.070	0.162±0.050★	0.249±0.062	0.248±0.072
52	0.213±0.072	0.168±0.052★	0.255±0.063	0.255±0.075
53	0.220±0.074	0.173±0.056★	0.261±0.066	0.261±0.081
54	0.225±0.071	0.181±0.059★	0.268±0.067	0.267±0.078
55	0.230±0.070	0.188±0.063	0.275±0.070	0.273±0.075
56	0.236±0.067	0.193±0.061★	0.283±0.069	0.280±0.068
57	0.245±0.064	0.203±0.060★	0.291±0.067	0.285±0.058
58	0.260±0.058	0.220±0.059★	0.299±0.062	0.291±0.050
59	0.274±0.052	0.241±0.060	0.306±0.056	0.296±0.046
60	0.287±0.048	0.264±0.060	0.310±0.049	0.298±0.045
61	0.296±0.044	0.283±0.051	0.312±0.045	0.301±0.046
62	0.296±0.042	0.292±0.046	0.310±0.040	0.298±0.047
63	0.294±0.040	0.296±0.042	0.304±0.036	0.291±0.044
64	0.287±0.038	0.293±0.041	0.296±0.033	0.281±0.041
65	0.277±0.038	0.286±0.039	0.284±0.031	0.269±0.038
66	0.268±0.040	0.275±0.038	0.270±0.033	0.256±0.033
67	0.258±0.041	0.263±0.038	0.257±0.034	0.247±0.030
68	0.249±0.040	0.252±0.037	0.247±0.034	0.239±0.028
69	0.242±0.036	0.243±0.035	0.241±0.033	0.232±0.027
70	0.238±0.033	0.237±0.032	0.239±0.031	0.228±0.026
71	0.235±0.030	0.236±0.029	0.238±0.029	0.223±0.024
72	0.232±0.029	0.235±0.028	0.237±0.028	0.219±0.022
73	0.230±0.029	0.237±0.029	0.237±0.028	0.215±0.020
74	0.229±0.027	0.239±0.029	0.235±0.030	0.212±0.020
75	0.230±0.026	0.244±0.031	0.233±0.033	0.213±0.020
76	0.234±0.026	0.249±0.031	0.229±0.033	0.215±0.022
77	0.238±0.026	0.256±0.032	0.226±0.034	0.219±0.025
78	0.245±0.024	0.267±0.034★	0.228±0.036	0.224±0.029
79	0.256±0.025	0.283±0.041★	0.236±0.039	0.232±0.037
80	0.271±0.027	0.304±0.049★	0.250±0.040	0.246±0.045
81	0.291±0.031	0.327±0.056★	0.270±0.041	0.263±0.053
82	0.316±0.041	0.354±0.063★	0.292±0.046	0.284±0.058
83	0.347±0.050	0.385±0.067★	0.320±0.049	0.308±0.062
84	0.383±0.057	0.423±0.068	0.353±0.053	0.336±0.066
85	0.421±0.063	0.461±0.068	0.387±0.058	0.365±0.069
86	0.454±0.062	0.495±0.064★	0.422±0.059	0.395±0.068
87	0.483±0.057	0.521±0.057★	0.457±0.058	0.421±0.065
88	0.509±0.055	0.543±0.051	0.491±0.058	0.448±0.064^?^
89	0.533±0.055	0.562±0.049	0.520±0.058	0.466±0.065^?^
90	0.546±0.053	0.574±0.046	0.537±0.059	0.476±0.061^?^
91	0.546±0.054	0.570±0.046	0.544±0.060	0.484±0.059^?^
92	0.545±0.055	0.562±0.048	0.551±0.062	0.489±0.062^?^
93	0.542±0.059	0.554±0.051	0.556±0.065	0.486±0.066^?^
94	0.538±0.063	0.546±0.059	0.556±0.068	0.498±0.065^?^
95	0.548±0.056	0.552±0.054	0.565±0.063	0.515±0.063^?^
96	0.557±0.050	0.560±0.050	0.574±0.058	0.533±0.063
97	0.567±0.047	0.569±0.047	0.585±0.054	0.556±0.066
98	0.608±0.035	0.606±0.048	0.625±0.043	0.604±0.065
99	0.657±0.030	0.654±0.053	0.673±0.036	0.654±0.067
100	0.707±0.040	0.701±0.066	0.720±0.038	0.703±0.071

p<0.05, showing the significant difference between the ICGs from the elderly subjects’ free gait and those from the young subjects’ free, fast and slow gait;

p<0.05, showing the significant difference between the ICGs from the young subjects’ fast gait and those from the young subjects’ free, slow gait and the elderly subjects’ free gait;

p<0.05, showing the significant difference between the ICGs from the young subjects’ slow gait and those from the young subjects’ free, fast gait and the elderly subjects’ free gait. T-TEST uses the two-tailed distribution, two-sample unequal variance (heteroscedastic).

**Figure 6:**
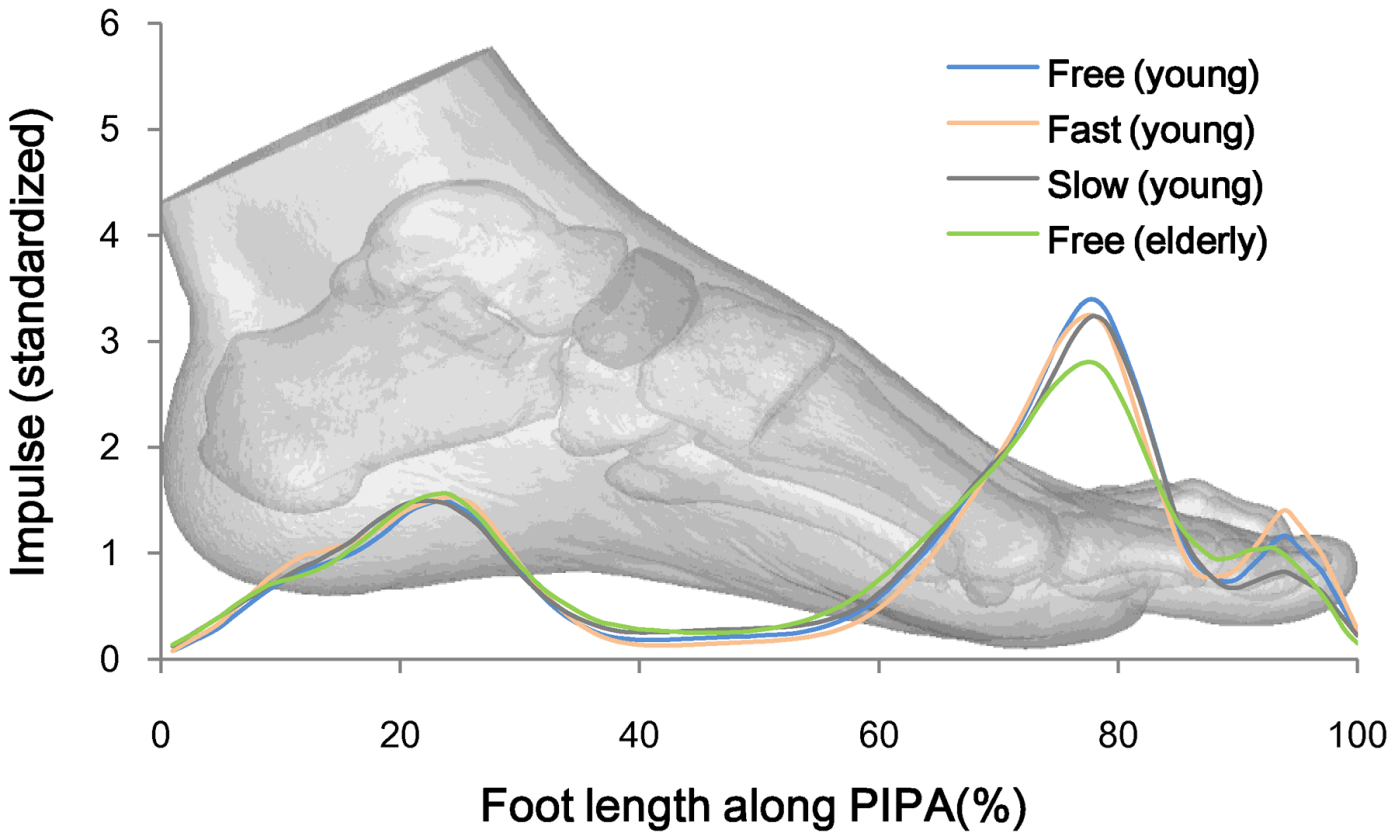
The impulse distribution along foot length.

Table 2 shows that a toe’s (1st toe) ICG time series index is created along PIPA, where the elderly subjects’ index is significantly different from that of the young at different walking speeds while for the young subjects, no significant difference can be spotted at different walking speeds. Why did this happen? We use Equation (6) to calculate plantar-impulse distribution along foot length. The mean value of impulse from each subject’s six continuous plantar impulses along foot length is drawn. See Figure 6.

Figure 5C shows that the interaction between the metatarsal and the ground happens at the terminal of stance phase, and its anteroposterior friction is forward [21], which keeps the walking speed. Figure 6 shows that when the young walk faster, their first toe plantar impulses increase. It is known that the aging of muscular system weakens the functions of the metatarsal. To make up for the decline in muscle strength [22], it is essential to increase the action time to enhance the interaction between the metatarsal and the ground because an impulse is a vector quantity given by the force over time. The reaction time between the elderly metatarsal and the ground in Figure 5D serves as an example. When the stance time is normalized, the increase of this reaction time is achieved by an earlier ICG.

Injuries and aging of the human musculoskeletal systems can cause changes to the gait parameters at walking speed [23], [24]. But these gait parameters may not be used to predict when injury information will fade or disappear nor when aging information will emerge. For example, ATR reconstruction can return to normal after 24 months [25]. But our research results can tell that ATR information always can be identified no matter how long ago an ATR happened. Walking speed is related to elderly subjects’ health [25]–[27], but we cannot examine their health just by walking speed because some elderly subjects just walk slowly: this might be their habit. The index that we created can reflect the difference between the elderly subjects’ toe PRTS at their free gait with that from the young subjects at their free, fast and slow gait, which suggests that the walking plantar impulse can serve as an index to analyze and evaluate musculoskeletal system injuries and aging.

Limitations of this research include: 1) if the angle formed by the connection line between the first intersection point and the second intersection point has something to do with the ATR, why is the angle formed from the healthy subjects not zero? Is this caused by the difference in the function of an individual’s left and right foot? A new gait experiment should be designed to explain this phenomenon.; 2) the reason why the elderly subjects’ ICG time series of the toe is different from that from the young subjects is not clear. Further research is needed to explain whether this phenomenon is caused by the musculoskeletal or neural system. In addition, though the receiver operating characteristic (ROC) curve cannot tell the incidence of the disease being tested, it serves as an effective method to evaluate the diagnostic test sensitivity [28]. In our future study, we will apply ROC curves to determine the clinical value and validity of our method.

### Conclusion

Basic gait parameters such as stride length and speed have been widely applied to make clinical diagnoses and to evaluate rehabilitation. Interesting and scientific results have been reached to eliminate individuals’ differences by applying these indexes such as stride length and hip height. For example, to estimate dinosaur’s walking speed by its stride length and hip height [29], [30]. The walking plantar impulse may not be an exception.

The standardization of the plantar impulse involves many quantities – plantar-impulse progression angle, size, plantar pressure, and plantar impulse. Contributions from Keijser and other scientists have been enlightening [14]. In our study, an analytical solution is applied to identify PIPA. The method to standardize the plantar impulse based upon the PIPA has guaranteed the accuracy. To the sensor, PRTS might serve as a logical data type as well as a time quantity. (*Specifically, a certain position of foot contacts the ground in a stance phase while it does not contact the ground in another stance phase. “To have” or “not to have” is a logical quantity. To such a logical record quantity, we cannot say that in successive walking, this position does not contact the ground; neither can we calculate its mean value directly.*) On the plantar impulse that has been fixed by PIPA, a calculation method based upon PRTS along PIPA can solve the algebraic operation problem for this quantity.

Walking is a moving process when the foot interacts with its support surface. In this process, when the movement of body segments changes, so does its interaction because it is the result of the interaction between objects. Musculoskeletal system injuries and aging have changed the movement of body segment [31]–[33]. In turn, the walking plantar impulse can reflect the status of musculoskeletal system injuries and aging. Indexes PRTS along PIPA and distribution of plantar impulse are applied to analyze ATRs, elderly and young subjects’ plantar impulse. The results verify that this works.

In conclusion, we can use PIPA to locate and standardize plantar impulse, and we can use the located and standardized plantar impulse to create indexes of PRTS and plantar-impulse distribution along PIPA. If this assessment index is validated by large size samples, this plantar-impulse analysis method may be widely used in clinical and rehabilitation medicine.

## Supporting Information

Table S1 **Basic parameters of gait from the young and the elderly subjects.**
(DOC)

Table S2 **Basic parameters of gait from the ATRs.**
(DOC)

File S1 **Walking plantar-impulse principal axis.**
(XLS)

## References

[1] Orlin MN, McPoil TG (2000) Plantar pressure assessment. Phys Ther 80: 399–409. 10758524 10.1093/ptj/80.4.399

[2] Pfeiffer M, Kotz R, Ledl T, Hauser G, Sluga M (2006) Prevalence of flat foot in preschool-aged children. Pediatrics 118: 634–639. 16882817 10.1542/peds.2005-2126

[3] Titianova EB, Mateev PS, Tarkka IM (2004) Footprint analysis of gait using a pressure sensor system. J Electromyogr Kines 14: 275–281. 10.1016/S1050-6411(03)00077-4 14962780

[4] Leeden MVD, Steultjens M, Dekker JHM, Prins APA, Dekker J (2006) Forefoot joint damage, pain and disability in rheumatoid arthritis patients with foot complaints: the role of plantar pressure and gait characteristics. Rheumatology (Oxford) 45: 465–469. 16287922 10.1093/rheumatology/kei186

[5] Bus SA, Van Deursen RWM, Kanade RV, Wissink M, Manning EA, et al (2009) Plantar pressure relief in the diabetic foot using forefoot offloading shoes. Gait Posture 29: 618–622. 19217785 10.1016/j.gaitpost.2009.01.003

[6] Najafi B, Crews RT, Armstrong DG, Rogers LC, Aminian K, et al (2010) Can we predict outcome of surgical reconstruction of Charcot neuroarthropathy by dynamic plantar pressure assessment - A proof of concept study Gait Posture. 31: 87–92. 10.1016/j.gaitpost.2009.09.003 19836956

[7] Wrobel JS, Najafi B (2010) Diabetic foot biomechanics and gait dysfunction. J Diabetes Sci Technol 4: 833–845. 20663446 10.1177/193229681000400411 PMC2909514

[8] Flaherty JH, Liu ML, Ding L, Ding BR, Ding QF, et al (2007) China: the aging giant. Am Geriatr Soc 55: 1295–1300. 10.1111/j.1532-5415.2007.01273.x 17661972

[9] Olshansky SJ, Goldman DP, Zheng Y, Rowe JW (2009) Aging in america in the twenty-first century: Demographic forecasts from the macarthur foundation research network on an aging society. Milbank Q 87: 842–862. 20021588 10.1111/j.1468-0009.2009.00581.x PMC2888016

[10] Lim BO, Lee YS, Kim JG, An KO, Yoo J, et al (2009) Effects of sports injury prevention training on the biomechanical risk factors of anterior cruciate ligament injury in high school female basketball players. Am J Sports Med 37: 1728–1734. 19561174 10.1177/0363546509334220

[11] Verhagen EA, Van Stralen MM, Van Mechelen W (2010) Behaviour, the key factor for sports injury prevention. Sports Med 40: 899–906. 20942507 10.2165/11536890-000000000-00000

[12] Giacomozzi C, Macellari V (1997) Piezo-dynamometric platform for a more complete analysis of foot-to-floor interaction. IEEE Trans Rehabil Eng 5: 322–330. 9422457 10.1109/86.650285

[13] Bennett MR, Harris JWK, Richmond BG, Braun DR, Mbua E, et al (2009) Early hominin foot morphology based on 1.5-million-year-old footprints from Ileret, Kenya. Science 323: 1197–1201. 19251625 10.1126/science.1168132

[14] Keijsers NLW, Stolwijk NM, Nienhuis B, Duysens J (2009) A new method to normalize plantar pressure measurements for foot size and foot progression angle. J Biomech 42: 87–90. 19056086 10.1016/j.jbiomech.2008.09.038

[15] Keijsers NLW, Stolwijk NM, Pataky TC (2010) Linear dependence of peak, average, and pressure–time integral values in plantar pressure images. Gait Posture 31: 140–142. 19800795 10.1016/j.gaitpost.2009.08.248

[16] Van Zee EH, Minstrell J (1997) Using questioning to guide student thinking. The Journal of the Learning Sciences 6: 229–271.

[17] Abe S (2008) Instability of q-averages in nonextensive statistical mechanics. EPL 84: 60006.

[18] Fan Y, Fan Y, Li Z, Lv C, Zhang B (2012) Bone Surface Mapping Method. PLoS ONE 7: e32926. 22412952 10.1371/journal.pone.0032926 PMC3297603

[19] Christopher LV, Brian LD, Jeremy CO (1999) *Dynamics of human gait*. Kiboho Publishers, Cape Town, South Africa.

[20] Andriacchi TP, Ogle JA, Galante JO (1977) Walking speed as a basis for normal and abnormal gait measurements. J Biomech 10: 261–268. 858732 10.1016/0021-9290(77)90049-5

[21] Riener R, Rabuffetti M, Frigo C (2002) Stair ascent and descent at different inclinations. Gait posture 15: 32–44. 11809579 10.1016/s0966-6362(01)00162-x

[22] Bischoff-Ferrari HA, Dietrich T, Orav EJ, Hu FB, Zhang Y, et al (2004) Higher 25-hydroxyvitamin D concentrations are associated with better lower-extremity function in both active and inactive persons aged≥60 y. Am J Clin Nutr 80: 752–758. 15321818 10.1093/ajcn/80.3.752

[23] Kang HG, Dingwell JB (2008) Separating the effects of age and walking speed on gait variability. Gait Posture 27: 572–577. 17768055 10.1016/j.gaitpost.2007.07.009

[24] Stanaway FF, Gnjidic D, Blyth FM, Le Couteur DG, Naganathan V, et al (2011) How fast does the grim reaper walk? Receiver operating characteristics curve analysis in healthy men aged 70 and over. Brit Med J 343: d7679. 22174324 10.1136/bmj.d7679 PMC3240682

[25] Don R, Ranavolo A, Cacchio A, Serrao M, Costabile F, et al (2007) Relationship between recovery of calf-muscle biomechanical properties and gait pattern following surgery for Achilles tendon rupture. Clin Biomech (Bristol, Avon) 22: 211–220. 10.1016/j.clinbiomech.2006.10.001 17126970

[26] Bendall MJ, Bassey EJ, Pearson MB (1989) Factors affecting walking speed of elderly people. Age Ageing 18: 327–332. 2603841 10.1093/ageing/18.5.327

[27] Dangour AD, Albala C, Allen E, Grundy E, Walker DG, et al (2011) Effect of a nutrition supplement and physical activity program on pneumonia and walking capacity in Chilean older people: a factorial cluster randomized trial. Plos Med 8: e1001023. 21526229 10.1371/journal.pmed.1001023 PMC3079648

[28] Everitt BS (2006) The Cambridge Dictionary of Statistics. Cambridge University Press, New York.

[29] Alexander RMcN (1976) Estimates of speeds of dinosaurs. Nature 261: 129–130.

[30] Alexander RMcN (2006) Dinosaur biomechanics. Proc R Soc B 273: 1849–1855. 10.1098/rspb.2006.3532 PMC1634776 16822743

[31] Kannus P, Sievanen H, Palvanen M, Jarvinen T, Parkkari J (2005) Prevention of falls and consequent injuries in elderly people. Lancet 366: 1885–1893. 16310556 10.1016/S0140-6736(05)67604-0

[32] Zeni JA, Higginson JS (2009) Differences in gait parameters between healthy subjects and persons with moderate and severe knee osteoarthritis: A result of altered walking speed? Clin Biomech 24: 372–378. 10.1016/j.clinbiomech.2009.02.001 PMC2715920 19285768

[33] Pandy MG, Andriacchi TP (2010) Muscle and joint function in human locomotion. Annu Rev Biomed Eng 12: 401–433. 20617942 10.1146/annurev-bioeng-070909-105259

