# Supplementary material for: Screening Method Based on Walking Plantar Impulse for Detecting Musculoskeletal Senescence and Injury
Source: PLoS One. 2013 Dec 30;8(12):e83839. doi: 10.1371/journal.pone.0083839 (PMC3875488; doi:10.1371/journal.pone.0083839)
Supplement: Table S1 — Basic parameters of gait from the young and the elderly subjects. (DOC) [file pone.0083839.s001.doc]

|  | **Normal (*n*=20)** | **Fast (*n*=20)** | **Slow (*n*=20)** | **Elderly (*n*=30)** |
| --- | --- | --- | --- | --- |
| Step length, left, cm | 67.15±6.10^◇^ | 78.26±6.71^◇^ | 60.00±5.26 | 59.18±5.15 |
| Step length, right, cm | 67.12±6.62^◇^ | 77.83±7.33^◇^ | 59.98±6.03 | 59.69±4.88 |
| Step time, left, sec | 0.51±0.02^◇^ | 0.43±0.03^◇^ | 0.59±0.06^◇^ | 0.54±0.04 |
| Step time, right, sec | 0.51±0.03^◆^ | 0.42±0.03^◇^ | 0.59±0.06^◇^ | 0.54±0.04 |
| Stance phase, left, % | 61.92±1.46 | 59.50±1.59^◇^ | 63.90±1.41^◆^ | 62.77±1.70 |
| Stance phase, right, % | 62.26±1.29^◆^ | 59.98±1.58^◇^ | 63.93±1.57 | 63.30±1.74 |
| Swing phase, left, % | 38.08±1.46 | 40.50±1.59^◇^ | 36.10±1.41^◆^ | 37.23±1.70 |
| Swing phase, right, % | 37.74±1.29^◆^ | 40.02±1.58^◇^ | 36.07±1.57 | 36.70±1.74 |
| Stride length, cm | 134.67±12.64^◇^ | 156.38±14.09^◇^ | 119.82±11.21 | 119.18±9.86 |
| Stride time, sec | 1.03±0.05^◇^ | 0.85±0.06^◇^ | 1.19±0.12^◇^ | 1.08±0.08 |
| Cadence, st/min | 58.51±2.70^◆^ | 70.85±5.46^◇^ | 51.08±4.55^◇^ | 56.00±4.29 |
| Speed, m/sec | 1.31±0.14^◇^ | 1.84±0.19^◇^ | 1.02±0.15^◆^ | 1.11±0.14 |

^◇^p<0.01, ^◆^p<0.05. T-TEST uses the two-tailed distribution, two-sample unequal variance (heteroscedastic).
