# Supplementary material for: Screening Method Based on Walking Plantar Impulse for Detecting Musculoskeletal Senescence and Injury
Source: PLoS One. 2013 Dec 30;8(12):e83839. doi: 10.1371/journal.pone.0083839 (PMC3875488; doi:10.1371/journal.pone.0083839)
Supplement: Table S2 — Basic parameters of gait from the ATRs. (DOC) [file pone.0083839.s002.doc]

|  | **Normal (*n*=7)** | **Fast (*n*=7)** | **Slow (*n*=7)** |
| --- | --- | --- | --- |
| Step length, left, cm | 64.53±3.05 | 79.86±4.42^◇^ | 57.87±5.58^◆^ |
| Step length, right, cm | 64.06±2.04 | 81.00±3.93^◇^ | 57.12±5.73^◆^ |
| Step time, left, sec | 0.53±0.06 | 0.45±0.03^◇^ | 0.60±0.07 |
| Step time, right, sec | 0.53±0.06 | 0.45±0.04^◇^ | 0.60±0.09 |
| Stance phase, left, % | 62.94±2.45 | 59.12±1.70^◇^ | 63.58±2.12 |
| Stance phase, right, % | 62.46±1.57 | 60.08±1.42^◇^ | 63.44±1.35 |
| Swing phase, left, % | 37.06±2.45 | 40.88±1.70^◇^ | 36.42±2.12 |
| Swing phase, right, % | 37.54±1.57 | 39.92±1.42^◇^ | 36.56±1.35 |
| Stride length, cm | 128.86±4.94 | 161.10±7.82^◇^ | 115.26±11.38^◆^ |
| Stride time, sec | 1.06±0.12 | 0.90±0.07^◇^ | 1.20±0.16 |
| Cadence, st/min | 57.33±5.42 | 67.41±5.42^◇^ | 51.09±6.18 |
| Speed, m/sec | 1.23±0.13 | 1.81±0.21^◇^ | 0.98±0.18^◇^ |

^◇^p<0.01, ^◆^p<0.05. T-TEST uses the two-tailed distribution, two-sample unequal variance (heteroscedastic).
